# Retroperitoneal Solitary Fibrous Tumor

**DOI:** 10.5334/jbsr.2150

**Published:** 2020-07-02

**Authors:** Jin Ho Seo, Seung Soo Kim, Woong Hee Lee

**Affiliations:** 1 Department of Radiology, Soonchunhyang University College of Medicine, Cheonan Hospital, Cheonan-si, Chungcheongnam-do, KR

**Keywords:** solitary fibrous tumor, retroperitoneal space, multidetector computed tomography, magnetic resonance imaging

## Abstract

**Teaching Point:** The typical imaging features of a retroperitoneal solitary fibrous tumor are relatively large-sized, have a well-defined margin, an avidly heterogeneous enhancement on arterial phase, a prolonged enhancement on delayed phase, and serpentine vessels along the periphery of the tumor.

## Case History

**Figure 1:**
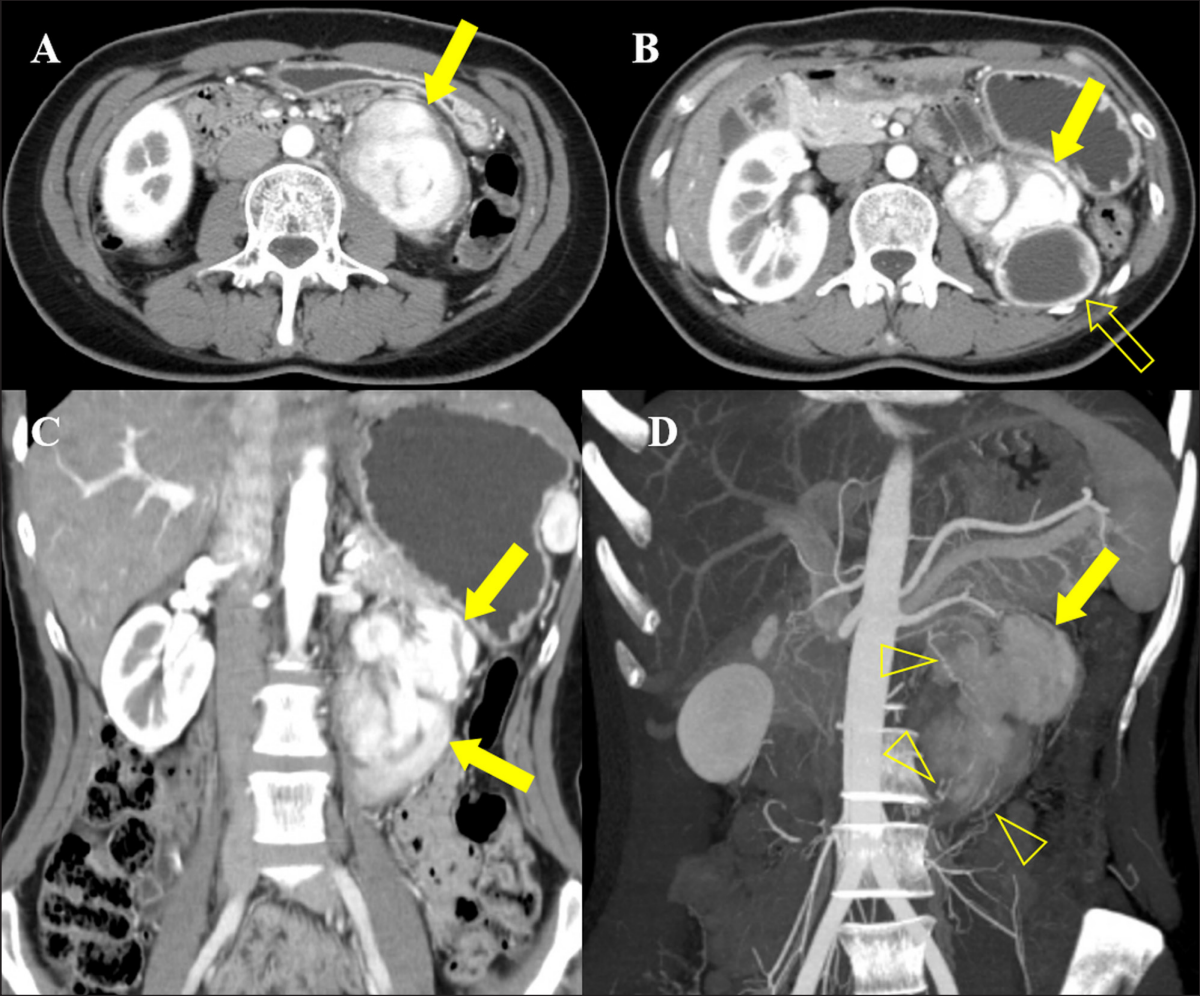


**Figure 2:**
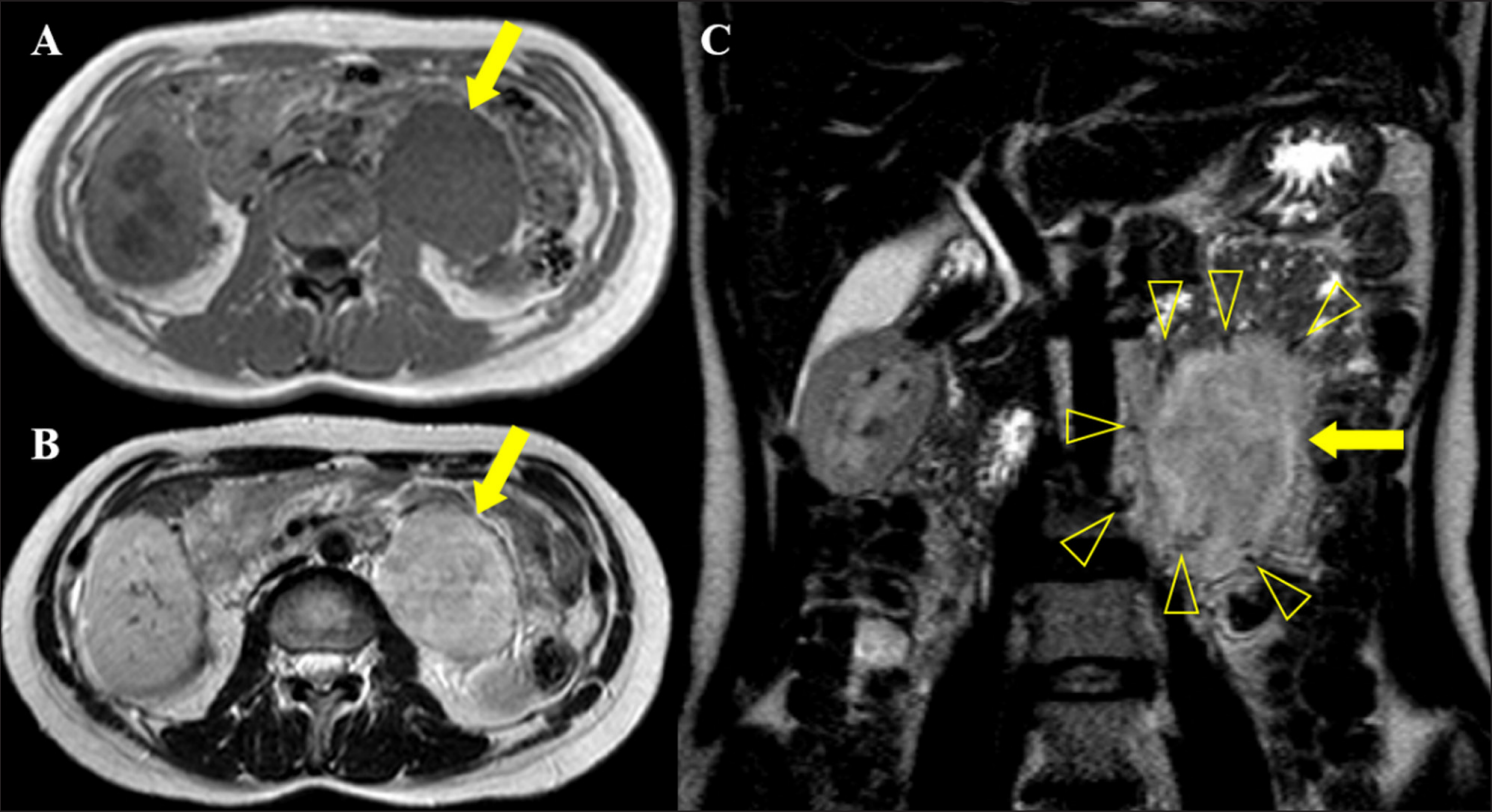


**Figure 3:**
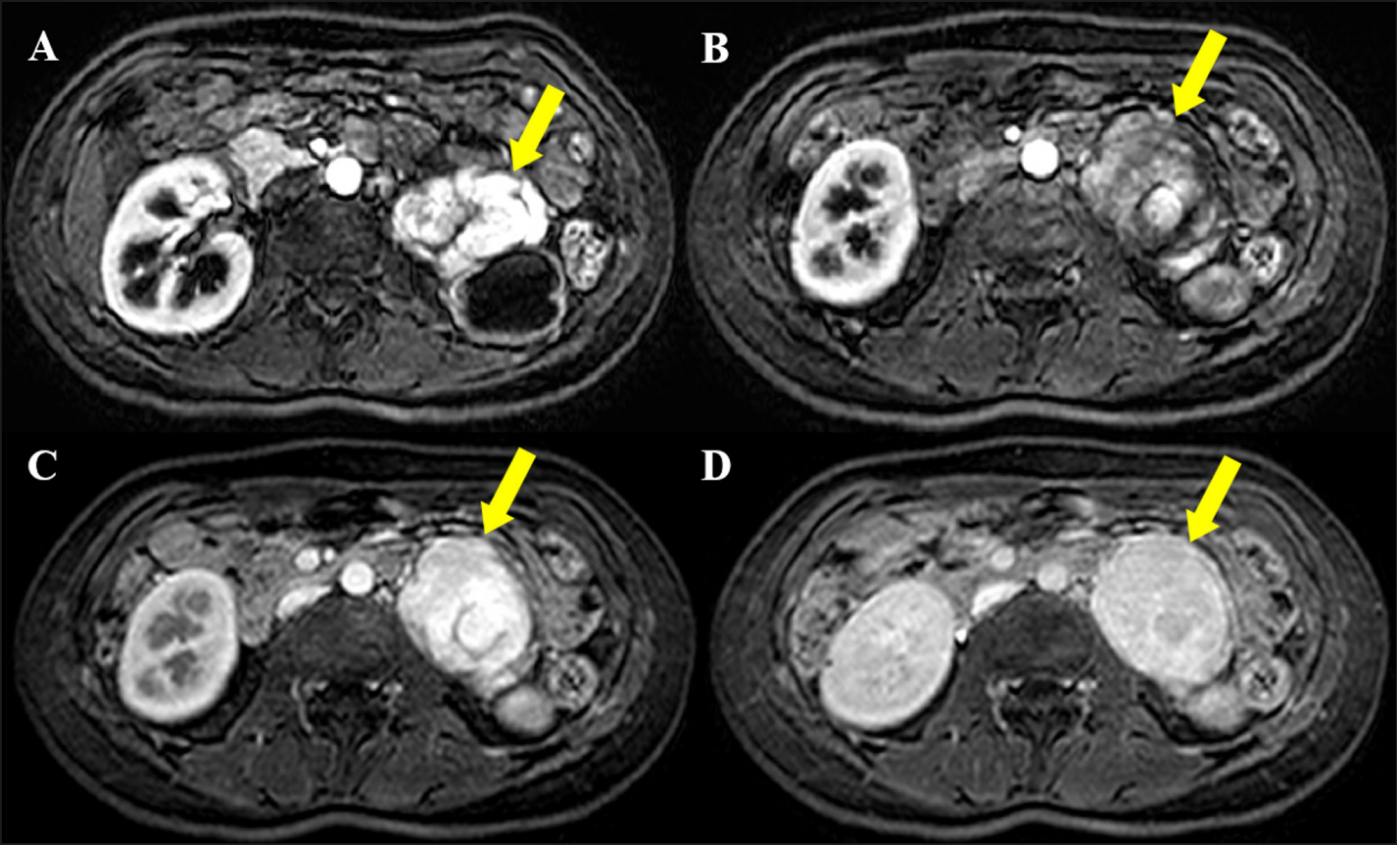


A 30-year-old female was admitted to our hospital after an elevated carbohydrate antigen (CA 19-9, 42.1 U/ml) was discovered on a health check-up. Her past medical history was unremarkable. Contrast-enhanced computed tomography (CT) was performed to check for abdominal malignancy. Axial and coronal reformatted arterial phase CT images (Figure 1A, B, C) showed an 8 cm, avidly heterogeneous enhancing mass (arrow) below the left kidney (open arrow). Left hydronephrosis (open arrow) occurred as the mass (arrow) compressed the left upper ureter. Coronal maximum-intensity projection reformatted CT image (Figure 1D) revealed multiple serpentine arteries (open arrowheads) along the periphery of the mass (arrow). The patient underwent magnetic resonance imaging (MRI) for further evaluation. The retroperitoneal mass (arrow) was iso-intense on T1-weighted image and hyperintense on T2-weighted image, with a well-circumscribed margin (Figure 2A and 2B). Coronal T2-weighted image revealed multiple signal voids (open arrowheads) along the periphery of the mass (arrow), a finding that suggests the vessels (Figure 2C). Axial dynamic gadolinium-enhanced T1-weighted images demonstrated the mass (arrow) with heterogeneous and strong enhancement on the arterial phase (Figure 3A and 3B) and prolonged enhancement on the portal venous and three-minute delayed phase (Figure 3C and 3D). The patient underwent mass excision and left nephrectomy, and she was diagnosed with retroperitoneal solitary fibrous tumor (SFT).

## Comment

SFTs are rare spindle-shaped cell tumors that account for less than 2% of all soft-tissue tumors [1]. They are considered to be ubiquitous neoplasm of fibroblastic or myofibroblastic origin. SFT is frequently detected in middle-aged adolescents, with equal distribution among the sexes. Although visceral pleura is the most common site of SFT occurrence, more than one-third of SFTs occur outside of the thoracic cavity, including the spine, mediastinum, pericardium, head and neck, and abdominal cavity. These tumors usually manifest as a slow-growing, asymptomatic mass. Most SFTs have a benign clinical course, but 10–15% of these tumors reveal an aggressive nature with recurrence or metastasis [1].

Less than 100 cases of retroperitoneal SFTs have been reported in the English literature, with a mean size of approximately 9 cm [1]. Some retroperitoneal SFTs are diagnosed in patients with hypoglycemia due to insulin-like growth factor II that is secreted by the tumor cells. On contrast-enhanced imaging, retroperitoneal SFTs typically show intense enhancement on the arterial phase and persistent enhancement on the delayed phase. In addition, serpentine vessels develop along the periphery of the retroperitoneal SFT. Retroperitoneal SFTs usually reveal a well-defined margin, and compress rather than invading adjacent organs. Hemorrhage, necrosis, or cystic degeneration can occur in retroperitoneal SFTs, but calcification is rare. The differential diagnosis for retroperitoneal SFT observed on an image includes neurogenic tumor, leiomyosarcoma, desmoid tumor, and undifferentiated pleomorphic sarcoma. However, pre-operative image diagnosis is not possible because of the rarity of retroperitoneal SFT. Complete surgical resection is the treatment of choice [1].
